# A 12-Week Electronic Mentoring Employment Preparation Intervention for Youth With Physical Disabilities: Pilot Feasibility Randomized Controlled Trial

**DOI:** 10.2196/12088

**Published:** 2019-03-29

**Authors:** Sally Lindsay, Elaine Cagliostro, Joanne Leck, Jennifer Stinson

**Affiliations:** 1 Bloorview Research Institute, Holland Bloorview Kids Rehabilitation Hospital Department of Occupational Science & Occupational Therapy University of Toronto Toronto, ON Canada; 2 Bloorview Research Institute Holland Bloorview Kids Rehabilitation Hospital Toronto, ON Canada; 3 Telfer School of Management University of Ottawa Ottawa, ON Canada; 4 Hospital for Sick Children Bloomberg Faculty of Nursing University of Toronto Toronto, ON Canada

**Keywords:** social support, mentor, employment, youth, disabled persons, rehabilitation, occupational therapy

## Abstract

**Background:**

Youth with disabilities are at high risk of unemployment compared with youth without disabilities. They often encounter challenges in accessing vocational programs that meet their needs. One promising approach that could help to address barriers that youth encounter while also enhancing social support is through electronic mentoring (e-mentoring). Although there is an increase in e-mentoring for youth with disabilities, little is known about its impact for youth with physical disabilities.

**Objective:**

This study aimed to assess the acceptability and initial impact of a Web-based peer electronic mentor employment intervention for youth with physical disabilities.

**Methods:**

The *Empowering Youth Towards Employment* intervention was evaluated using a pilot randomized controlled trial (RCT). Youth, aged 15-21 years, with physical disabilities were randomly assigned to an intervention (ie, mentored) or control (ie, not mentored) group. Trained mentors (ie, near peers) with a physical disability led the online discussion forums and provided peer support and resources for 12 modules (1 topic per week over 12 weeks). Primary outcomes focused on self-determination, career maturity, and social support. We also explored program adherence and dosage, participant satisfaction, and areas for improvement.

**Results:**

A total of 13 youth (mean age 17.3 years, SD 1.88; 54%, 7/13 female) completed the RCT. In the intervention group (n=9), 56% (5/9) of the youth were females, and in the control group (n=4), 50% (2/4) of the youth were female. Participants reported satisfaction with the program and that it was feasible and acceptable. Participants’ mean engagement level with the program was 5.22 (SD 2.48) for the intervention group and 5.40 (SD 4.56) for controls. Participants in the intervention group demonstrated significant improvements in self-determination (*t*_12_=2.49; *P*<.04) compared with the control group. No adverse events were reported.

**Conclusions:**

The *Empowering Youth Towards Employment* is a promising intervention that enhances self-determination among youth with physical disabilities.

**Trial Registration:**

ClinicalTrials.gov NCT02522507; https://clinicaltrials.gov/ct2/show/NCT02522507 (Archived by WebCite at http://www.webcitation.org/6uD58Pvjc)

**International Registered Report Identifier (IRRID):**

RR2-10.2196/resprot.8034

## Introduction

### Background

Although many young people with disabilities are willing and capable of working, they encounter many personal, environmental, and socio-contextual challenges (eg, inaccessible jobs and discrimination) in finding and maintaining meaningful employment [1]. Their persistently low employment rates are about half or less compared with youth without disabilities [2-4]. Although youth with disabilities could benefit from employment training programs, they are often not tailored to meet the needs of youth with disabilities (eg, self-care at work, disclosing a disability, and requesting accommodations). Of the programs that are targeted toward people with disabilities, they often focus on youth with intellectual or developmental disabilities, whereas less attention has been paid to youth with physical disabilities [1,3,4]. There are limited evidence-based employment preparation programs for youth with disabilities in Canada [5,6]. For example, a systematic review by Hanif et al [5] focusing on employment preparation programs for youth with physical disabilities found only 8 empirical studies in this area. Although there is limited research on this particular population, the findings are promising and show potential to improve self-confidence, self-awareness, goal setting, and knowledge of career options [5]. Although some evidence suggests that vocational programs can influence employment outcomes for youth with disabilities, much further research is needed [5]. Focusing on youth with physical disabilities is important because they arguably have different needs regarding developmental tasks, social development, and role functioning [7,8]. Furthermore, this period of emerging adulthood is an optimal time to help youth develop critical job and independence skills [9].

Mentoring involves developing a relationship between a more experienced individual who serves as a role model and shares knowledge with a less experienced individual [10,11] and can help provide youth with informational, practical, and emotional assistance to enhance coping skills as youth transition to adulthood [12-15]. Mentoring has beneficial impacts on job training, educational attainment, social skills, self-esteem, self-efficacy, work ethic, and employment outcomes [16-18]. Mentoring is particularly useful for groups that are considered to be disadvantaged, such as youth with disabilities [15,19]. Research on mentoring programs among youth without disabilities shows that they are a cost-effective way to augment vocational and educational services while also promoting positive behaviors (eg, self-efficacy, quality of life, and employment skills) [12,20-23]. A meta-analysis focusing on youth without disabilities showed that the key ingredients of peer mentor interventions involve trained mentors, monitored implementation, structured activities, and parental involvement [20]. Until recently, most mentoring programs (in general and those specific to employment) have not included nor specifically targeted youth with disabilities [15,18]. A common challenge with mentoring programs is that it is often difficult to meet face-to-face. Thus, having a Web-based format can help to overcome some of these challenges [24]. Peer electronic mentoring (e-mentoring) may be 1 way to help youth to gain valuable employment preparation skills in an accessible format. Research demonstrates that Web-based platforms can influence learning and behavior change [25-27]. Web-based formats are particularly relevant for youth, given that the majority of them seek information and communicate over the internet [28]. E-mentoring formats are also flexible in regard to matching participants to a mentor and asynchronous communication [29].

### Rationale

This study addresses an important gap in the literature by offering a Web-based employment preparation intervention for youth with physical disabilities. Such youth often encounter different challenges compared with youth with invisible disabilities or chronic illnesses because their condition is often visible, and they also encounter difficulties in mobilities, speech, independence, coping, stigma, and social exclusion [30]. Our intervention aims to strengthen youth’s employment preparation skills including self-determination, career maturity, and social support, all of which can have beneficial effects for employment outcomes [15,19,31-34].

## Methods

### Objective

Our main objective was to assess the feasibility, acceptability, and initial efficacy (ie, pilot randomized controlled trial, RCT) of an electronic mentor (e-mentor) employment preparation intervention for youth with physical disabilities for improving self-determination, career maturity, and social support compared with controls. A secondary objective includes exploring program adherence and dosage, participant satisfaction, and areas for improvement.

### Design

A pilot RCT, with an embedded qualitative design [35], was chosen to test the feasibility and initial efficacy of the *Empowering Youth Towards Employment* intervention for youth with physical disabilities. This intention to treat design involves an intervention group that received employment preparation Web-based modules and a peer e-mentor. Meanwhile, the control group received the Web-based modules only (no mentor) but could interact with other participants within their group. We administered pre- and postsurveys (immediately following the completion of the intervention) for both groups (intervention and control). We followed the Medical Research Council Framework for the development and evaluation of RCTs to guide our design [36]. We focused on the development and feasibility phases to establish the theoretical underpinnings and modeling to test the feasibility of key intervention components [36]. The qualitative data comprised open-ended survey questions and researcher’s observation field notes.

The rationale, design, content, and length of our intervention was based on 2 systematic reviews focusing on employment preparation interventions for youth with physical disabilities [5] and best practices of peer mentorship for improving employment outcomes [15] and 2 scoping reviews on improving the inclusion of people with disabilities in the workforce [37] and mentoring practices for a diverse workforce [38]. We also conducted needs assessments regarding informational and social support for youth with disabilities [7,39].

### Procedures and Randomization

**Figure 1:**
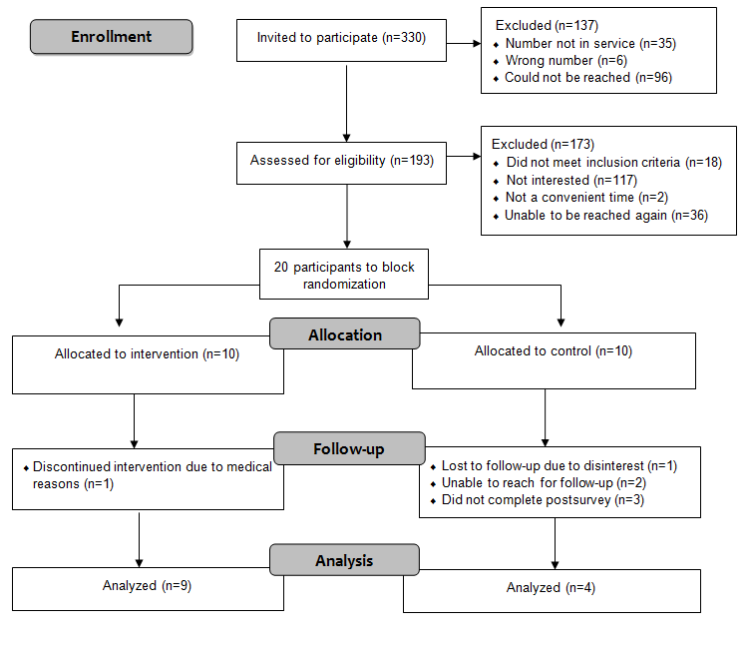
Search process flow diagram.

We received institutional research ethics board approval (from a pediatric hospital and a university). Eligible participants were sent an information letter and phone call from the research team. The research assistant screened all participants and obtained informed written consent before enrolling them in the intervention. Once participants consented, they were randomized into an intervention or control group using a block size of 10 [40]. Participants were then emailed the presurvey (see measures below). Next, a research assistant contacted participants to inform them of their group assignment and instruct them on the procedures to be followed (see Figure 1 for trial schema).

#### Intervention (Experimental Group)

The purpose of the intervention was to provide meaningful support and access to evidence-based employment resources so that youth can begin thinking about preparing for employment. The content and length of our intervention were evidence-informed by 2 systematic reviews, a scoping review, and a needs assessment conducted by our team [5,15,37,39]. It was cocreated with a knowledge user advisory group and consists of 12 modules (1 per week over 12 weeks) that were delivered by youth peer mentors in a password-protected online discussion forum (see Multimedia Appendix 1 for topics). Each module contained informative resources and interactive materials (ie, articles and videos) that could be viewed at their own pace, homework, and discussions led by trained peer mentors. The group-based intervention involved up to 10 participants per group plus 2 mentors (ie, trained near-peers who have a physical disability). Youth were given access to a separate password-protected area of *AbilityOnline* website, a safe forum for youth with disabilities. It was important to note that we piloted the 12-week format before switching to a 4-week format (reported elsewhere) [6].

Youth mentors presented each of the topics to mentored participants in the same order using a script. To ensure treatment fidelity, each mentor received the same training and regular check-ins with project staff. They provided their own personal experiences and examples related to each topic and were instructed to respond to all posts while offering informational, emotional, and social support, which they received training in. The asynchronous discussion forum was available to all participants in the group (eg, intervention or control). Participants were instructed to log in to the forum at least once a week, which they could do at a time that was convenient for them. Mentors posted their availability (ie, when they would be in the forum) if participants wanted to discuss something in real time. To be considered for the role of a peer mentor, eligible young adults had lived experience with a physical disability, some employment experience, and have completed the 3-day Youth Peer Mentor Training program that runs out of a pediatric rehabilitation hospital [41]. Mentors also completed project-specific training (ie, active listening, perspective taking, confidentiality, maintaining boundaries, positive role modeling, trust building through interactive training, and mentoring) before starting. Mentors included 2 youth (1 male and 1 female, mean age 21.5 years) who had a physical disability. Mentors introduced the topics in the same order and were trained to respond to participants’ comments in a similar supportive and positive-focused manner [6].

#### Control Group

The control group had access to a separate password-protected group within the Web-based forum that contained the modules only, and they did not receive peer mentorship [6]. Instead, a researcher posted the weekly discussion topics but was instructed to not reply to any participant posts. Youth participants within the control group could interact with other participants in their group, but such discussions were not facilitated by a mentor.

### Recruitment

Participants were recruited from June to September 2016 through invitation letters sent from a pediatric hospital and disability organizations through referrals and advertisements. Potential participants also contacted us if they were interested in participating. Inclusion criteria involved the following: (1) able to read and write in English, (2) aged 15 to 25 years, (3) have access to a computing device with internet access, (4) currently enrolled in or recently completed a high school diploma in the applied or academic stream (to screen for cognitive impairment), (5) have no paid work experience, and (6) youth with a physical disability (eg, cerebral palsy, spina bifida, muscular dystrophy, and spinal cord injury) [6]. Here, we refer to disability as impairment, activity limitation, and participation restriction, whereby a disability and functioning are shaped by interactions between health conditions and contextual factors [42]. Our rationale for this age group and also choosing youth without employment experience was that youth with disabilities often start their first job later than youth without disabilities [39]. Exclusion criteria involve those who recently completed or who are currently participating in another employment preparation or peer support intervention [6].

Information packages were mailed to 330 potential participants, and the research team followed up with potential participants via phone (n=298) or email (n=32) to assess interest and availability for the study. If youth were interested in participating in the study, they were then screened for eligibility by a researcher, and if eligible, they were required to sign a written consent or assent form before taking part in the study. We were able to reach and assess 193 participants for eligibility. A total of 173 participants were excluded (see Figure 1 for reasons). All the 13 participants who joined the study provided written consent, completed a presurvey, signed up for the intervention website, and completed the postsurvey after the completion of the intervention. A total of 20 participants expressed interest in the study, met the inclusion criteria, and provided written consent. Using a block size of 10, participants were randomly assigned into either the experimental (ie, mentored) or control group (see Figure 1 for trial schema).

A total of 7 participants dropped out of the study. Of the participants, 1 dropped out of the intervention group because of medical complications, and 6 participants dropped out of the control group (see Figure 1 for reasons). A total of 13 participants completed the intervention and postsurveys (9 intervention and 4 controls).

### Outcome Measures

In addition to basic demographic information (see Table 1), participants completed 3 standardized measures at baseline and at completion of the study to explore self-determination [43], career maturity inventory attitude scale [43], and multidimensional scale of perceived social support [44]. These standardized measures have good internal consistency, construct-related and criterion validity, and test-retest reliability and have been widely used for youth with disabilities [43-46]. Given that the focus of this study was feasibility, these outcome measures were exploratory to examine variances to help determine appropriate outcomes and sample size for future full-scale RCTs.

The first outcome measure, Arc’s Self-Determination Scale [46], consists of a self-report measure that assesses self-determination for adolescents with disabilities, with subscales on autonomy, acting on the basis of preferences and abilities (postschool directions), goal setting, and task performance (eg, “I make my own my own meals or snacks” and “I make long-range career plans”). The scale includes I do not even if I have the chance (0), I do sometimes when I have a chance (1), I do most of the time when I have a chance, and I do every time I have the chance (3) [46].

The second outcome measure, Career Maturity Inventory Attitude Scale [43,45], is a 25-item agree (1) or disagree (0) scale where responses form the bases for 5 subscales relating to career decision making including orientation, involvement, independence, compromise, and decisiveness (eg, “there is no point in deciding on a job when the future is so uncertain,” “I don’t know what courses I should take in school.” and “I keep changing my occupational choice”) [45,47].

The third measure, Multidimensional Scale of Perceived Social Support, includes a 12-item questionnaire that captures perceived social support from various sources (ie, parents, siblings, friends, and peers). Example items include “there is a special person who is around when I am in need,” “my family really tries to help me,” and “I can count on my friends when things go wrong.” The scale involves the following: very strongly disagree (0), strongly disagree (1), mildly disagree (2), neutral (3), mildly agree (4), strongly agree (5), and very strongly agree (6). Scores are summed for a total score, with higher scores reflecting higher values of social support [47].

Secondary measures include Web-based usage (ie, number of times logged in and length of time on the forum); how much they liked each topic, based on the Web-hosting *Drupal* platform analytics. We also describe adherence with the intervention, engagement (self-rated scale of 0-10, with higher scores reflecting higher engagement in the program), and satisfaction with the program as measured through open-ended questions in the postsurveys. Other parameters explored included recruitment accrual rates, program adherence, and satisfaction with the intervention, along with suggestions for improvement.

### Data Analysis

Data were analyzed using IBM SPSS, version 25. Descriptive statistics were used to describe the sample characteristics at baseline using means and SD for continuous variables and frequencies and portions for categorical variables. *t* tests were conducted to compare baseline characteristics between the intervention and control groups. Separate analyses were conducted for each outcome. Holm’s sequential correction will be applied to control for type I error. Effect sizes were determined using Cohen *d*, with 0.2 as indicative of a small effect, 0.5 medium effect, and 0.8 large effect. A level of .05 was used as the criterion for statistical significance.

Qualitative analysis of the open-ended survey questions and researcher’s field notes involved 2 team members reviewing all data independently, and then, they compared the findings and analyzed them thematically through an open-coding, constant comparison approach [48]. Any discrepancies were resolved through discussion of the themes and reevaluated until consensus was reached. We kept a log of the key decisions made throughout the analysis to help improve the credibility of our findings [48].

## Results

### Sample Characteristics

A total of 13 adolescents (mean age 17.3, SD 1.88; 54% female; range 15-21 years) completed the study, which ran from July 2016 to December 2016. A total of 6 participants had cerebral palsy, 4 had muscular dystrophy, 2 had Charcot Marie tooth disease, and 1 had metabolic bone disease. A total of 6 participants in the intervention group and 4 in the control group used an assistive or mobility device. The majority of the participants (6 intervention, 4 control) were currently enrolled in school. The remainder completed high school or were unemployed. There were no significant differences between the groups on demographic variables at baseline (see Table 1).

In regard to self-rated engagement in the intervention, there were no significant differences between the intervention (mean 5.22, SD 2.48) and control groups (mean 5.40, SD 4.56). Both groups spent a similar amount of time on the website (intervention: mean 1.1 hours, SD 1.3; control: mean 1.55 hours, SD 1.85).

**Table 1:** Demographic characteristics of participants and mentors.

Demographic characteristics		Participants		Mentors (n=2)
		Intervention (n=9)	Control (n=4)	
Age (years), mean (SD)		17.8 (1.7)	16.25 (1.89)	21.5 (1.5)
**Sex (n)**				
	Male	4	2	1
	Female	5	2	1
**Disability type (n)**				
	Cerebral palsy	4	2	1
	Muscular dystrophy	3	1	0
	Charcot Marie tooth disease	2	0	0
	Metabolic bone disease	0	1	0
	Other physical disability	0	0	1
Use an assistive or mobility device (n)		6	4	2
Currently enrolled in school (n)		6	4	2
Total time spent on the website (hours), mean (SD)		1.1 (1.3)	1.55 (1.85)	6.38 (5.41)
Number of posts and messages, mean (SD)		6.77 (6.49)	3.0 (4.69)	35.5 (6.36)
Number of logins, mean (SD)		34.3 (59.0)	7.25 (3.68)	99.5 (85.55)
Self-rated engagement, mean (SD)		5.22 (2.48)	5.40 (4.56)	7 (0)

Although the intervention group logged in more times (intervention: mean 34.3, SD 59.0; control: mean 7.24, SD 3.68) and posted more messages on average than controls (intervention: mean 6.77, SD 6.49; control: mean 3.0, SD 4.69), the differences between the groups were not significant.

### Primary Outcome Analysis

Our primary outcome for this study focused on the feasibility of the *Empowering Youth Towards Employment* intervention. *t* tests (between time 1 and time 2) were computed to examine differences on each outcome including self-determination, career maturity, and social support. Separate analyses were conducted for each outcome. There were no significant differences on outcome measures at baseline. After controlling for baseline scores, participants in the intervention group had significantly improved self-determination scores after the intervention compared with those in the control group (*t*_12_=2.49; *P*<.03; *d*=.70; see Tables 2 and 3 for comparison of outcome measures between the 2 groups). There were no significant differences in career maturity or social support between the intervention and control groups following the intervention.

#### Program Adherence and Dosage

Mentors delivered the intervention as instructed in the protocol and posted a unique topic once a week. The researchers monitored this through the discussion boards. The mentor’s role was also to respond to participant comments, which was done majority (82%) of the time. A total of 18% of the messages posted by participants did not get a response from mentors. A total of 2 of these messages were follow-up responses from participants on a specific topic. In a few instances, mentors posted a longer, more generic statement that addressed several different comments rather than addressing each specific participant.

The control group ran as planned, where a research assistant posted 1 topic per week, which included general information and discussion questions. The researcher who posted the topics in the control group did not respond to any of the participants’ posts in the forum, as directed in the protocol. All 12 topics were posted at the beginning of the week as prescribed, and the full extent of the intervention was delivered to participants.

#### Participant Satisfaction With the Intervention

A total of 8 out of 9 (89%) of intervention group participants and 4 out of 4 (100%) of the control group participants said they would recommend the intervention to others. In their open-ended survey responses, participants described their level of satisfaction with the intervention and whether they would recommend it to others. Specifically, youth in both the intervention and control groups reported satisfaction with receiving information and feedback. A total of 3 participants in the intervention and 3 in the control group reported enjoying the information provided in the intervention, especially the employment preparation content and disability specific resources. For example, a participant in the intervention group commented that the intervention “gives a lot of advice on how to get to work where you want to and how to overcome the disability, which is helpful” [#1-09]. Furthermore, giving and receiving feedback was another aspect that participants (5 intervention, 1 control) appreciated. Such advice included feedback from peer mentors related to career strategies, overcoming disability-related challenges, and sharing experiences related to the intervention topics. For example, 1 intervention group participant shared:

I was able to suggest some things I did that helped me with some of my issues which I hope helped my colleagues.

#1-03

Participants also expressed contentment with the Web-based format of the intervention, stating it was “well-organized” [#1-02] and that the Web-based format “was the easiest one to learn from and get information from” [#1-05]. Furthermore, 7 participants in the intervention group had positive feedback regarding the social interaction and social support components provided through the intervention. For example, a participant shared that they “enjoyed connecting with...the mentors because it made the ideas of transition to postsecondary seem less daunting”[#1-05]. Another youth highlighted a benefit of the program:

It is very important to hear the opinions of people with disabilities. So, if someone feels alone they know they’re not the only ones going through certain situations.

#1-02

**Table 2:** Descriptive statistics on participant outcomes (time 1) by group.

Variable	Intervention group (n=9), mean (SD)	Control group (n=4), mean (SD)	*t (df)*	*P* value	Effect size (Cohen *d*)
Self-determination	22.3 (5.17)	14.5 (3.69)	3.35 (12)	.06	0.88
Career maturity	13.0 (3.27)	13.0 (7.02)	0 (12)	>.99	0
Social support	49.5 (21.07)	58.0 (17.7)	−1.005 (12)	.33	0.21

**Table 3:** Differences in outcomes between experimental and control groups (time 2).

Variable	Intervention group (n=9), mean (SD)	Control group (n=4), mean (SD)	*t (df)*	*P* value	Effect size (Cohen *d*)
Self-determination	23.0 (5.78)	13.5 (7.59)	2.49 (12)	.03	0.7
Career maturity	13.75 (3.10)	15.25 (4.11)	−0.71 (12)	.49	0.71
Social support	49.5 (21.07)	60.5 (13.27)	−0.946 (12)	.36	0.32

One of the main goals of the intervention was to provide employment resources and information to participants, which was deemed as helpful by most participants, such as participant #1-04, who stated:

The study was very informative and helped a lot with my plans for my career.

A participant in the control group also discussed that they enjoyed the program because “you get good information and tips“ [#2-03].

#### Areas for Improvement

Some participants had suggestions about how the intervention could be improved. For example, some participants noted that it would be helpful to have more prompts to keep them engaged throughout the 12 weeks. Lack of engagement was often associated with personal reasons (ie, too busy or forgetting to log in, too shy to participate, etc). For instance, a participant in the intervention group explained:

I enjoyed the program; however my summer became very busy and I regret not being able to engage in the program.

#1-02

Another participant suggested:

Sometimes I forgot to check the website during the week so it would be helpful to receive an email reminding me to check any updates.

#1-03

One participant in the intervention group who had logged in few times but did not post describes:

I needed greater clarity on time frame and expectations. I’m sorry I was a passive participant and did not engage in the discussions more.

#1-01

A total of 3 intervention participants and 1 control group participant mentioned that they found it difficult to remember to log in to the website or had limited access to a computer, which made it difficult to participate on a regular basis.

Other participants wanted improvements to the website. For instance, a participant in the control group explained that they found the “layout of the website was confusing...and hard to navigate” [#2-05]. Meanwhile, a participant in the intervention group suggested:

The website could have a better UI (User Interface) so it has a better feel.

#1-09

## Discussion

### Principal Findings

Our findings show that the *Empowering Youth Towards Employment* intervention demonstrated acceptability and preliminary evidence of impact in 1 of the outcome measures within a sample of youth with physical disabilities. Youth with disabilities are considered a vulnerable population that has unique vocational needs [15]. Helping them to gain employment skills is important because they often encounter significantly higher unemployment rates compared with youth without disabilities [6,49]. Therefore, providing mentoring and resources within a Web-based forum is 1 potential way that can help to engage youth with disabilities within an accessible format [50]. Peer mentors can act as role models who help to normalize the experience of transitioning to work for those who have a disability. Knowing that others have gone through a similar experience may help to increase their motivation for pursuing vocational interests [51,52]. Youth may be more receptive to receiving information from a peer who is closer in age [52]. Previous research shows that mentoring is a promising mechanism that can help to enhance youth’s inclusion while also offering support and coping strategies [53]. Web-based platforms can also influence learning and behavior change [26]. Our study is novel in that it offers an employment preparation program through an e-mentor platform. Most previous studies focus on self-management and health-related outcomes.

Our results indicated significant improvements in self-determination (large effect) among the youth in the intervention (ie, mentored) group. Previous research shows that there is a strong link between mentoring and improvements in self-efficacy [54], self-determination [55,56], and self-confidence [57]. For example, Gregg et al’s [55] study explored the effectiveness of virtual mentoring to enhance the persistence of secondary and postsecondary students with learning disabilities in science, technology, engineering, and mathematics and found improved self-determination [55]. Indeed, helping youth to develop their self-determination and self-advocacy skills is important because it is critical for optimizing their participation and inclusion in society [58-60].

A total of 2 of the measures within our study, career maturity and social support, showed no significant improvement following the intervention. These findings are somewhat surprising and contrast other e-mentor interventions focusing on youth with visual impairments and blindness (using a mixed format including face-to-face and group-based activities, email, and phone calls) that found significant improvement in career decision making [61]. Another study involving 8 e-mentoring sessions to enhance the transition to college for youth with disabilities [62] found significant improvements in career decision self-efficacy. This discrepancy could be partly a result of the small sample size, differences in the disability types explored, the length of the mentoring interventions, differences in the measures used, and lack of control groups in these studies.

Research shows that early vocational experiences are associated with the development of career maturity and vocational identity, contributing to a positive career trajectory [33,34]. Vocational maturity is characterized by the extent to which one is concerned with seeking out a career choice; investigating and planning for an occupation; stable occupational preference over time; realistic attitudes toward work; and habits, interests, and abilities that match one’s occupational preference [33,63]. Most studies exploring career maturity among youth with disabilities focus on the college age [31]. Given that the average age of our sample was younger, it could be that youth are still very early on in their development and need more time and experience to further develop their career maturity.

Our results also showed no significant improvements in social support within the time frame of this study. This finding could be a result of the social support provided by peer mentors (eg, encouragement and sharing experiences), perhaps acting as a mediating factor affecting self-determination [32]. For example, research shows that employment success can be achieved through social relationships [64] or perhaps, the participants in our study already had good support systems in place from family and friends. Some research suggests that the e-mentoring process lacks verbal communication cues, which result in more impersonal or superficial relationships between mentor and mentee, and therefore, more informal or conversational language should be used to encourage closeness [53,65]. Interestingly, participants expressed satisfaction in social interactions and support from the intervention in the open-ended survey responses.

Our findings highlighted participants’ satisfaction with the intervention, and the majority would recommend it to other youth. Somewhat surprising, however, is that their self-reported levels of engagement were somewhat lower than expected. This could have been because of the 12-week length of the program and many youth were busy with school and often found it difficult to participate as often as they would have hoped to. We observed that engagement levels, based on the number of posts per topic, decreased over the course of the 12-week intervention. We recommend that the research team and youth mentors check in more regularly with the participants to help keep them engaged throughout the intervention. Other studies on mentoring among youth with disabilities show that having small group activities [66] and family support [21,67] can help keep participants engaged, along with timely responses from mentors [65].

Our results showed lower than expected engagement levels, which suggest that the intervention may not be appropriate in its current form for youth with physical disabilities. The intervention could benefit from flexibility in the length of the program to increase acceptability to a larger group of youth. Some youth may have found it difficult to remain engaged for 12 weeks, especially with ongoing school commitments. Our findings are consistent with past research focusing on youth without disabilities, showing that maintaining engagement of participants within an e-mentor format can be challenging, and engagement may decrease as the intervention progresses [68,69]. One topic per week may also not be enough interaction to keep youth engaged, as research shows that frequent and consistent contact is useful for a successful mentoring relationship [65,70,71]. Therefore, we recommend that a shorter, more condensed format, perhaps over the summer months when youth are not as busy with school, may be more suitable.

### Limitations and Future Directions

It is important to consider the limitations of this study, which focused on the feasibility and initial impact of the intervention. First, we were underpowered in some of the analyses. Our sample size was small, and we recruited from only 1 site, which limits the generalizability of the findings. Second, some youth who completed a presurvey and participated in the discussion forum did not complete a postsurvey, which excluded them from the analysis. This was particularly the case for the control group. Therefore, it is important to note that more of the participants in the intervention group stayed in the study. The design of future interventions should consider how to maintain engagement of participants who are in the control group. Third, we included various types of physical disabilities, and each have different needs and concerns regarding preparing for employment and could have affected the results. Fourth, we were unable to determine the number of participants who looked at the resources and modules and the amount of time that they spent looking at them, which is an important factor that could influence the outcomes that we explored. Finally, the host website that we used for this intervention went through several upgrades during the course of the intervention that caused some technical difficulties that some participants experienced. This could have affected their participation and engagement in the study.

Future studies should be directed in several areas. First, given the relatively low self-rated engagement levels, future research should have specific engagement strategies to keep participants engaged (in both the intervention and the control groups). This could involve regular check-ins to see if participants need any help with posting, reminders to contribute to the discussion, or involving a variety of different components such as face-to-face or in-person components [19]. Further support may be needed for participants who are shy or reluctant to post. Finally, further research should explore whether any potential gains from this or other similar interventions last over the long term.

### Conclusions and Implications

The *Empowering Youth Towards Employment* intervention is promising in that it enhances self-determination among youth with physical disabilities. There is a growing emphasis on the importance of including a process evaluation as part of an RCT [72]. This study shows that a process evaluation can provide valuable information about an intervention, which is critical if it going to be fully implemented into practice. Furthermore, findings from the process evaluation will help interpret the outcomes of the intervention. Our results show that the length of the intervention seemed too long for both the mentors and the participants. We recommend condensing the length of the intervention into a 1-month format that would also fit into the youth’s schedule. It is important to balance addr essing the primary mentoring goal of helping youth to develop employment-related skills with fostering a meaningful mentoring relationship with participants. We recommend that further mentor training and ongoing support is needed to help facilitate this. Furthermore, our results suggest that there is value in having peer mentors who have a disability. Most previous peer mentoring studies have a mentor that does not have a disability.

## Appendix

Multimedia Appendix 1 Overview of weekly topics.

Multimedia Appendix 2 CONSORT‐EHEALTH checklist (V 1.6.1).

## Abbreviations

e-mentor: electronic mentor
e-mentoring: electronic mentoring
RCT: randomized controlled trial
