# Supplementary material for: A 12-Week Electronic Mentoring Employment Preparation Intervention for Youth With Physical Disabilities: Pilot Feasibility Randomized Controlled Trial
Source: JMIR Pediatr Parent. 2019 Mar 29;2(1):e12088. doi: 10.2196/12088 (PMC6716483; doi:10.2196/12088)
Supplement: Multimedia Appendix 1 [file pediatrics_v2i1e12088_app1.pdf]

Multimedia Appendix 1: Overview of weekly topics

**Topic**
Introduction and goal setting
Life skills
Managing disability at work
Family role in supporting employment
Aspirations and expectations
Volunteerism
Finding a job
Social networking and community resources
Preparing for job interviews
Learning from professionals with disabilities
Career pathways and transitions
Referrals and next steps
